# Characterization of anode and anolyte community growth and the impact of impedance in a microbial fuel cell

**DOI:** 10.1186/s12896-014-0102-z

**Published:** 2014-12-09

**Authors:** Diana Sanchez-Herrera, Daniella Pacheco-Catalan, Ruby Valdez-Ojeda, Blondy Canto-Canche, Xochitl Dominguez-Benetton, Jorge Domínguez-Maldonado, Liliana Alzate-Gaviria

**Affiliations:** Renewable Energy Unit, Centro de Investigación Científica de Yucatán A.C (CICY), Calle 43 No. 130 Col. Chuburná de Hidalgo, C.P. 97200 Mérida, Yucatán México; Biotechnology Unit, CICY A.C, Mérida, Yucatán México; Separation and Conversion Technology, VITO–Flemish Institute for Technological Research, Boeretang 200, Mol, 2400 Belgium

**Keywords:** Microbial fuel cell, Community growth and electrochemical impedance spectroscopy

## Abstract

**Background:**

A laboratory-scale two-chamber microbial fuel cell employing an aerated cathode with no catalyst was inoculated with mixed inoculum and acetate as the carbon source.

Electrochemical impedance spectroscopy (EIS) was used to study the behavior of the MFC during initial biofilm (week 1) and maximum power density (week 20). EIS were performed on the anode chamber, biofilm (without anolyte) and anolyte (without biofilm). Nyquist plots of the EIS data were fitted with two equivalent electrical circuits to estimate the contributions of intrinsic resistances to the overall internal MFC impedance at weeks 1 and 20, respectively.

**Results:**

The results showed that the system tended to increase power density from 15 ± 3 (week 1) to 100 ± 15 mW/m^2^ (week 20) and current density 211 ± 7 (week 1) to 347 ± 29 mA/m^2^ (week 20). The Samples were identified by pyrosequencing of the 16S rRNA gene and showed that initial inoculum (week 1) was constituted by *Proteobacteria* (40%), *Bacteroidetes* (22%) and *Firmicutes* (18%). At week 20, Proteobacterial species were predominant (60%) for electricity generation in the anode biofilm, being 51% Rhodopseudomonas palustris. Meanwhile on anolyte, *Firmicutes* phylum was predominant with Bacillus sp.

This study proved that under the experimental conditions used there is an important contribution from the interaction of the biofilm and the anolyte on cell performance. Table 1 presents a summary of the specific influence of each element of the system under study.

**Conclusions:**

- The results showed certain members of the bacterial electrode community increased in relative abundance from the initial inoculum. For example, Proteobacterial species are important for electricity generation in the anode biofilms and *Firmicutes* phylum was predominant on anolyte to transfer electron.
- R1 is the same in the three systems and no variation is observed over time.
- The biofilm makes a significant contribution to the charge transfer processes at the electrode (R2 and Cdl) and, consequently, on the performance of the anode chamber.
- The biofilm can act as a barrier which reduces diffusion of the anolyte towards the electrode, all the while behaving like a porous material.
- The anolyte and its interaction with the biofilm exert a considerable influence on diffusion processes, given that it presents the highest values for Rd which increased at week 20.

## Background

Wastewaters contain dissolved organics that require removal before discharge into the environment. However, wastewaters are being recognized as a renewable resource for the production of electricity, fuels and chemicals. Bioelectrochemical wastewater treatment has, therefore, emerged as a potentially interesting technology for the production of energy [1]. Reducing the cost of the materials used in MFCs is essential for practical applications. The cathode accounts for the greatest percentage of the total capital cost, and cathode surface area and materials generally limit higher power production in MFCs. Therefore, it is important to identify low-cost materials and efficient cathode architectures in order to improve MFC cost effectiveness and performance [2].

The bioanode, a crucial component in bioelectrochemical systems (BESs), is composed of an anode biofilm and a conductive electrode. The main catalytic components of interest in anode biofilms are exoelectrogens, microorganisms that are capable of exocellular electron transfer [3,4]. In mixed-culture systems, exoelectrogens compete for electron donors with other functional groups such as fermenters, acetogens and methanogens [5,6]. The complexity of anode biofilms makes it hard to elucidate electrochemical mechanisms at the bioanode, but a precise understanding of exoelectrogenesis and competition in anode biofilms will aid in improving the performance of BESs. Several reviews provide insightful summaries and perspectives regarding bioanodes [7,8].

Electrochemical impedance spectroscopy (EIS) was used as a non-intrusive tool to identify and elucidate the electrochemical properties of redox mediators produced by microbes. EIS enabled the study of the individual contributions from different resistances. These include ohmic resistance (representing the resistance from solution, electrode materials and membrane), charge transfer and concentration (diffusion) resistance on the anode and cathode behavior of mediators and their impact on MFC impedance without the need to interrupt MFC operation [9,10].

With the help of equivalent electrical circuit fitting analysis, EIS can also provide quantitative estimates of the kinetic rate constants for the anodic and cathodic reactions, double layer capacitance at the electrode surface, and the diffusion coefficients of electro-active species in the bulk electrolyte [10,11].

The aim of this study was to characterize community growth on the anode and in the anolyte and the impact of electrochemical impedance in a microbial fuel cell with an initial mixed inoculum and its selection process over time.

## Results and discussion

### Chemical oxygen demand

COD removed in the first week was 93%. Meanwhile, in week 20 it was 86.5% due to increased planktonic cell concentration in the anolyte increasing the organic load and decreasing COD over time. This is similar to what Biffinger *et al.* [12] observed. The COD removed is found in the medium-high range (63% to 98%) of results obtained by other authors in the literature [13].

VFA concentrations at week 20 were 0.01 mM, 0.003 mM and 0.0009 mM for butyric, propionic and acetic acid, respectively. These values were insignificant with respect to the initial concentration of the electron donor at 73.14 mM of sodium acetate. The ease with which acetate was metabolized by exoelectrogenic microorganisms was also observed by Velasquez-Orta *et al.* [14], who obtained carboxylic acid concentrations of less than 0.05 mM after 30 weeks. The percentages of H_2_ and CH_4_ in the samples analyzed at week 20 of this study were less than 0.01%. This rules out a methanogenic biochemistry with a Coulombic efficiency of 60.6%. These values were lower than those reported by Jung and Reagan [8] in a PEM-type MFC with Pt catalyst at the cathode and acetate as the carbon source. They obtained 6% H_2_ and CH_4_ with a Coulombic efficiency of 6.4%.

### Composition by pyrosequencing

The Shannon index of diversity (H’) was determined for all samples. The H’ value was higher for “initial inoculum” (4.5) than “biofilm” (2.8) and “anolyte” (2.9). This indicates, that the initial inoculum was higher diverse than the other samples. The bacterial richness by Chao estimate indicated that initial inoculum showed a higher number of species (339) than the biofilm (142) and anolyte (150) samples, as was indicated by Shannon index.

**Figure 1:**
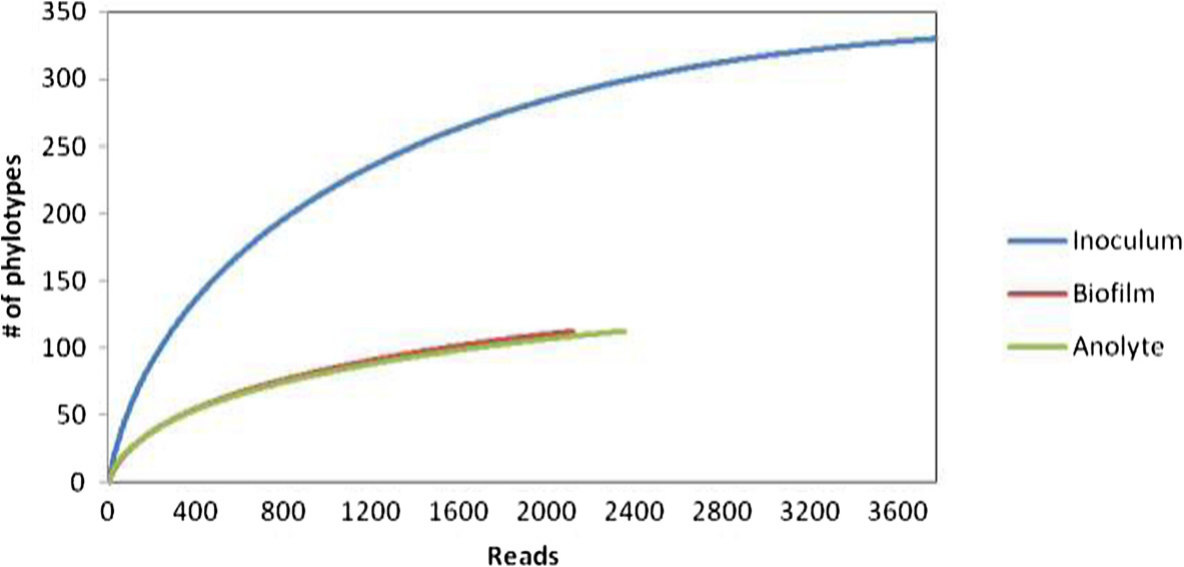
**Rarefaction curves for each sample from MFC.** Number of reads is shown on the x axis and number of OTUs at 95% sequence identity on the y axis.

The rarefaction curves (Figure 1) indicated higher OTU (Operational Taxonomic Unit) number on initial inoculum sample (330) than in biofilm (123) and anolyte (123). In comparison to initial inoculum curve, the curve of biofilm and anolyte samples is flatter with increasing sampling effort, and therefore possesses lower bacterial species.

#### Initial inoculum (1 week)

**Figure 2:**
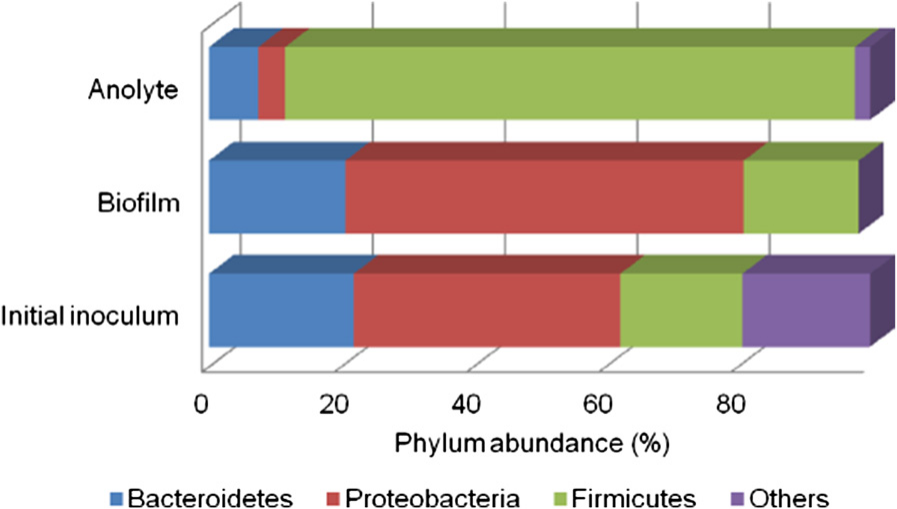
**Taxonomic classification of pyrosequences from predominant bacterial communities of initial inoculum, biofilm, anolyte of MFC at the phylum level.**

A total of 3,764 bacteria were detected in this sample. The phylogenetic spectrum (Figure 2) was dominated by *Proteobacteria* (40%), *Methylobacter* sp. (15%) and *Syntrophus* sp. (6%) as the predominant species. Within *Bacteroidetes* (22%), *Cytophagia order* (9%) was dominant with *Firmicutes* (18%) and *Clostridiales* family (5%). *Proteobacteria* phylum has been widely detected in microbial fuel cell studies. For example, in floating microbial fuel cell (FMFC), a modification of MFC the bacterial community was dominated by γ- and β-*Proteobacteria* (with identity to *Methylobacter luteus* species) [15]. Bacterioidetes [16] such as *Firmicutes* [17] have been frequently detected as dominant in MFC reactors. Primary clarifier effluents sampled from MFC showed phylotypes relatively abundant in *Proteobacteria, Bacteroidetes* and *Firmicutes* phyla [16].

#### Biofilm (without anolyte - 20 weeks)

A total of 2,121 bacteria were detected in the sample collected from the electrode. The most abundant were *Proteobacteria* (60%), followed by *Bacteroidetes* (21%) and *Firmicutes* (17%). *Bacteroidete* identification in biofilm is consistent with many reports indicating its presence (Zhang *et al.* [18]; Zhang *et al*. [19]). This suggests it may play a critical role in electricity generation or efficient anode function (Yusoff *et al*. [20]). It is very interesting to note that of the 60% of *Proteobacteria* found 51% of these bacteria corresponded to *Rhodopseudomonas palustris* species. This bacterium is a member of the phototrophic purple non-sulfur bacteria which proliferates in different environments due to its versatile metabolism. It is photoautotrophic, photoheterotrophic, chemoheterotrophic and chemoautotrophic [21]. Its capability in electricity generation has been reported before in R. *palustris* DX-1 by Xing *et al.* [22]. This DX-1 strain can produce higher power densities when isolated than as mixed cultures in the same MFC. The genome completes of CGA009 strain of R. *palustris* have been sequenced [21]. U.S. Department of Energy has anticipated that genome sequence comparisons between DX-1 and strains of R. *palustris* will probably reveal key biochemical characteristics of strain DX-1 that are critical for its ability to generate power (Figure 2).

#### Anolyte (without biofilm - 20 weeks)

A total of 2,349 bacteria were detected in the sample taken from electrode. *Firmicutes* (86%) predominated in this sample, followed by *Bacteroidetes* (7%), *Proteobacteria* (4%) and *Lentispharaerae* (2%) (Figure 2). Predominance of *Firmicutes* at week 20 (140 days) is in accordance with studies by Ishii *et al.* [23]. The dominance of *Firmicutes* phylum was been reported in MFC at 79% in current production with acetate as the electron donor [17]. *Thermincola* sp. was the predominant species that generated current independent of an electron shuttle with acetate as an electron donor. In this study, *Bacillus* species for *Firmicutes* phylum were represented at 80%. In this respect, Nimjea *et al.* [24] demonstrated that the aerobic Gram-positive species *Bacillus subtilis* was able to grow anaerobically and produce a biofilm in a microbial fuel cell which generated a long-term power output. The electrochemical activity and the electron transfer mechanism were mainly due to excreted redox mediators in the broth solution and not to the membrane-bound proteins which were affected by physiological status.

Numerous reports indicate that *Firmicutes* are integral members of the MFC bacterial community, indicating their exocellular electron transfer (Choo *et al*. [25]; Rabaey *et al.* [26]). Thus, they dominated in the anolyte sample because of dependent electrode respiration (maybe by redox gradient-driven c-type cytochromes).

### Power and current density

The maximum power and current densities were 100 ± 15 mW/m^2^ and 347 ± 29 mA/m^2^, respectively, at week 20. Values reported in the literature range from 80 to 1,330 mW/m^2^ and from 0.55 mA/m^2^ to 538 mA/m^2^ in MFCs with no catalyst at the cathode. The open circuit potential stabilized at 704 mV (data not shown) [1,27,28].

### Electrochemical impedance spectroscopy (EIS)

The EIS measurement of an individual electrode provides information that permits the analysis of electrochemical reactions on electrodes and bacterial metabolism, as well as surface and material properties of electrodes. These are critical to understanding the electricity-generating process and improving the power output of MFCs [29].

#### Anode chamber (Biofilm and Anolyte)

**Figure 3:**
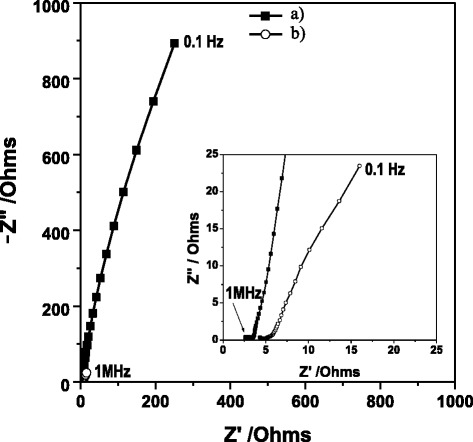
**Nyquist diagrams corresponding to anode chamber at different times of growth: a) week 1 and b) week 20.**

From Figure 3, it is clear that at both week 1 and week 20 an ohmic resistance (R1) is presented, followed by a semicircle at high frequencies, which corresponds to charge transfer processes on the surface of the electrode. The semicircle is characteristic of a single “time constant” [27]. Subsequently, a predominantly capacitive linear segment is observed (with medium to low frequencies) with a slight inclination. It is more evident in the diagram corresponding to week 20 due to the presence of other associated processes such as diffusion and/or charge transfer corresponding to the substrate. Generally, the bioelectrochemical substrate oxidation processes are slow, offer high impedance and are exhibited in the mid-to-low frequency domains [10]. For week 1, the charge transfer process of substrate oxidation is the rate limiting step since it is significantly slower than the mediator charge transfer process and oxygen reduction steps. The microbial growth on the anode has a beneficial effect on the kinetics of the bio-electrochemical reaction as it decreases the anode activation losses due to increased biocatalyst density [10]. Likewise, it is clear that a lower impedance response was present at week 20. If we consider impedance to be a vector with a specific magnitude [11], then from Figure 3 there is an improved performance in the anode chamber at week 20 (55.73 Ω) given that the impedance values are lower than week 1 (7,472 Ω) due to the presence of a biofilm. This development is similar to the report by Borole *et al*. [30] used a consortium enriched in a compact, flow-through porous electrode chamber coupled to an air-cathode. Anode impedance initially decreased from 296.1 Ω on day 24 (3.4 weeks) to 2.6 Ω after 6 months (24 weeks). Ramasamy *et al*. [10] reported that the anode impedance from two-chamber MFC on day 1 and week 3 were estimated to be 174 Ω and 32 Ω, respectively, indicating that the growth of the microbial biofilm was found to decrease the anode polarization resistance and facilitate the kinetics of the electrochemical reactions. For this study, the following elements were considered due to the similarity of the behavior of the Nyquist diagrams in accordance with what Bisquert *et al.* [31] reported. Both a Warburg element (W1) and anomaly diffusion (M_a_) were used to evaluate diffusion resistance (Rd) in terms of two parameters: Y_0_ and B. Y_0_ is the magnitude of the admittance at ω = 1 rad/s while B characterizes the time it takes for a reactant to diffuse through a thin fi w. The ratio B/Y_0_ indicates the magnitude of diffusion resistance [31].

On analyzing the values obtained, the ohmic resistance (R1) was not observed to present significant changes between week 1 (R1 = 19.38 ± 5.48 Ωcm^2^) and week 20 (R1 = 31.36 ± 9 Ωcm^2^). This contrasted with the resistance values corresponding to the charge transfer phenomena in which a reduction was observed due to the presence of the biofilm (week 1, R2 = 66,860 ± 3.89 Ωcm^2^; week 20, R2 = 434 ± 0.44 Ωcm^2^). Given its high conductivity, it facilitated the mobility of electrons through the electrode/biofilm interface. This behavior is similar to what Manohar *et al.* [9] found, as well as other authors such as Malvankar *et al*. [32] and Borole *et al.* [30].

CPE values for week 1 and 20 were 1.58 E-03 ± 5.7 E-06 Ω^−1^s^α^ and 0.08 ± 1 Ω^−1^s^α^ with α = 0.9, respectively. In both systems, the incorporated CPE is considered to be a non-ideal capacitor. For determination, it was considered a faradaic system with time-constant interfacial capacitance RC [11].

1 $$ \mathrm{C}={\left[\mathrm{Q}{\left(\frac{1}{\mathrm{R}1} + \frac{1}{\mathrm{R}2}\right)}^{\left(\upalpha -1\right)}\right]}^{1/\upalpha} $$

Q (S s^α^) is the admittance when $\omega =1,\kern0.5em j=\sqrt{-1}$; α is an ideal constant and R1 and R2 are the ohmic and charge transfer resistances, respectively.

**Figure 4:**
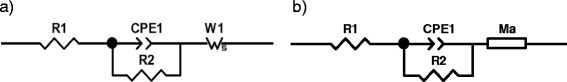
**Equivalent circuits used for the analysis of impedance data for the three systems: anode chamber, biofilm and anolyte at a) week 1 and b) week 20.**

On comparing the capacitance values between week 1 Cdl = 8.70 E-04 ± 1.03 E-10 F and week 20 Cdl = 8.37 E-02 ± 4.68 E-02 F, an increase in capacitance was observed by two orders of magnitude due to the fact that the biofilm facilitates charge accumulation at the electrode interface. Although the values may vary based on the experimental conditions (electrode material, inoculum type, etc.) [33], the behavior is similar to that observed in previous works. For example, Borole *et al*. [30] reported their values of Cdl in the anode of 0.01 F during the first two months (8 weeks). Also, it increases by two orders of magnitude from 61 to 136 days (19.4 weeks), obtaining a value of 0.42 ± 0.04 F. Also, Ramasamy *et al*. [10] reported values for double layer capacitance from 0.5 mF (day 1) to 0.9 mF (day 12) for a surface of 15 cm^2^ in anode, which is expected capacitance or carbon electrode. This confirmed that Cdl increased due to the stabilization of the system. As for the diffusion processes, these can occur when the solution species are diffused through the biofilm to the surface of the electrode [31]. At week 1, they are represented as a Warburg element (W1) (Figure 4) with linear, semi-infinite diffusion behavior (W1 = 6,485 ± 3,491.1Ω s^1/2^) equivalent to Rd = 360.25 ± 19.95 Ωcm^2^. At week 20, meanwhile, this element is replaced by a modified anomalous diffusion element (M_a_) (Figure 4) equivalent to a linear transmission model reported by Bisquert *et al*. [31] with Rd = 36.18 ± 7.7 Ωcm^2^. This decrease in resistance is caused by proton exchange due to the presence of the biofilm.

The model includes these elements, given that the impedance in the diffusion processes is defined as [34]:

2 $$ Z\left(i\omega \right)\propto {\left(i\omega \right)}^{-\beta /2}\left(0<\beta <2\right) $$

where, in the majority of cases, it is a Warburg-type impedance (β = 1). Anomalous diffusion (β ≠ 1) is characterized by a mean squared displacement of the diffusing particles that does not follow the ordinary linear law r^2^ ∝ t but, more generally, has a power law dependence on time: r^2^ ∝ t^β^ at low frequencies. The reason for this is the frequency transient time for a diffusing particle injected at x = 0 to cover a distance L. For ω > > ω_d_ the particles will not sense the boundary at x = L so that the system will behave as semi-infinite.

The presence of the biofilm promotes anomalous diffusion processes, i.e. protonic diffusion which can be spatially restricted in either planar, cylindrical or spherical forms, producing variation in the limits of the diffusion region 0 < x < L present at low frequencies.

Many different mechanisms give rise to this anomalous behavior, including complex flows, structural complexity in the substrate of diffusion and in the diffusing tracers. Thus, no single theory of anomalous diffusion can account for all possible phenomenologies as in this case for the presence of biofilm.

#### Biofilm (without anolyte)

**Figure 5:**
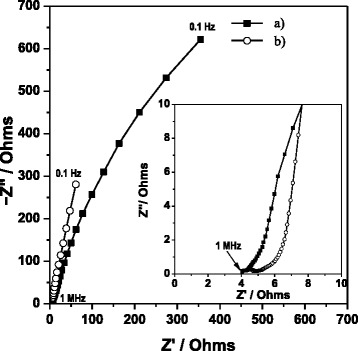
**Nyquist diagrams corresponding to biofilms at different times of growth: a) week 1 and b) week 20.**

A more common “coating layer” on the electrodes of MFCs is a biofilm. Figure 5 shows the Nyquist diagram of the biofilm at weeks 1 and 20. First of all, there is a difference between the two systems at the same frequencies with week 20 presenting lower impedance, once again considering impedance to be a magnitude vector. This is due to the fact that at week 20 the biofilm had already achieved growth and stabilization, favoring processes which reduce the total resistance of the system [30].

Likewise, at both weeks 1 and 20, the final part of a semicircle is observed, corresponding to charge transfer processes on the surface of the electrode (high frequencies) followed by a linear segment with an incline angle that corresponds to diffusion processes, and finally a predominantly capacitive linear portion similar to the behavior presented by the anode chamber. Therefore, the equivalent circuits proposed for the anode chamber at weeks 1 and 20 were used (Figure 4).

On analyzing the data obtained, ohmic resistance was not found to present significant changes between week 1 (R1 = 36.00 ± 4.12 Ωcm^2^) and week 20 (R1 = 35.99 ± 4.11 Ωcm^2^). With respect to the CPE, as in the case of the anode chamber, the behavior is mainly non-ideal capacitance. The values obtained (Cdl) at weeks 1 and 20 were 10.71E-01 ± 1.37E-03 F and 8.14 E-04 ± 1.48 E-10 F, respectively. This allows for the inference that the capacitance contribution in the anode chamber can be attributed to the behavior of the biofilm as has been reported previously by other research groups [30].

The charge transfer resistance for week 1 presented higher values (R2 = 24,651 ± 799.38 Ωcm^2^) compared to those at week 20 (R2 = 2,305.8 ± 12.4E-03 Ωcm^2^) due to the interaction of the biofilm with the anolyte.

Diffusion processes at weeks 1 and 20 were equivalent to Rd 32.51 ± 7.5 Ωcm^2^ and 32.22 ± 8.93 Ωcm^2^. These values were similar to those for the anode chamber at week 20, suggesting that the anolyte presents diffusion processes from the outset.

#### Anolyte (without biofilm)

**Figure 6:**
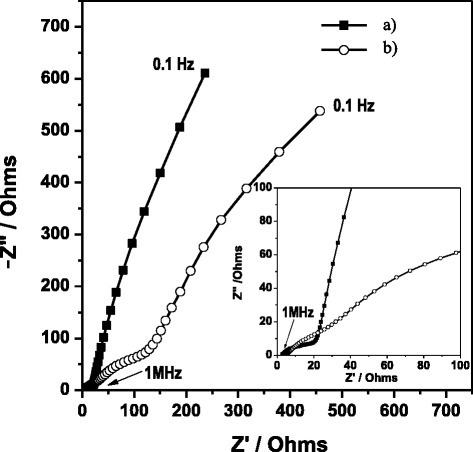
**Nyquist diagrams corresponding to anolyte at different times of growth: a) week 1 and b) week 20).**

Figure 6 shows the Nyquist diagram corresponding to the anolyte at weeks 1 and 20. In both cases, it was not possible to observe a variation in the impedance value, considering it to be a vector. The ohmic resistance is followed by an incomplete semicircle which indicates the presence of charge transfer processes. It is unlikely that any biochemically derived redox compounds, synthesized mediators, yield a complete faradaic response to an AC signal faster than 100 Hz. Hence, the reaction in the high frequency region depicts a fast electrochemical process such as oxidation of soluble metal ions in the growth medium.

The ohmic resistance values (R1) do not present significant changes due to the fact that they correspond to the resistance of the components of the system itself (weeks 1 and 20, 24.5 ± 15.55 Ωcm^2^ and 42.9 ± 3.92 Ωcm^2^, respectively).

The charge transfer resistance and diffusion resistance for week 1, R2 = 199 ± 3.89 Ωcm^2^ and Rd = 389.3 ± 33.50 Ωcm^2^, compared to week 20, R2 = 179 ± 47.8 Ωcm^2^ and Rd = 3.75E + 03 ± 1.37E-03 Ωcm^2^, show that the R2 values are similar, meaning that there is no contribution from the anolyte to the charge transfer processes. The anolyte without biofilm on the electrode is not redox-active toward acetate under these conditions. EIS of bare electrodes showed a very high charge transfer resistance [35].

When comparing the results of Rd obtained for the anolyte with those for the anode chamber and the biofilm, the anolyte at week 1 presents similar values to those for the anode chamber. However, at week 20 the value increases by an order of magnitude, whilst in the anode chamber it decreases by an order of magnitude because the diffusion resistance decreased considerably due to the presence of the biofilm.

CPE values for weeks 1 and 20 were 1.22E-03 ± 9.02E-05 (α = 0.6) and 1.40E-03 ± 1.18 E-03 (α = 0.6), corresponding to the capacitances (Cdl) 1.70E-05 ± 2.23E-08 F and 2.86E-05 ± 9.74E-06, respectively.

Although there is evidence in the literature for the presence of diffusion processes in this type of bioelectrochemical system indicating that the mass transfer limitations were insignificant and masked by the dominant kinetic limitations for the anode bio-electrochemical reaction, this study proved that under the experimental conditions used there is an important contribution from the interaction of the biofilm and the anolyte on cell performance. Table 1 presents a summary of the specific influence of each element of the system under study (A = highly influential; B = influential and C = uninfluential).

**Table 1:** **Influence of each of the elements corresponding to the phenomena in the MFC**

**Configuration**	**System**	**Charge transfer processes**		**Diffusion processes**
	**R1**	**R2**	**Cdl**	**Rd**
**Anode chamber**	**C**	**A**	**A**	**B**
**Anolyte**	**C**	**C**	**C**	**A**
**Biofilm**	**C**	**A**	**A**	**B**

## Conclusions

The Microbial Fuel Cell with no catalyst (granular carbon and stainless steel mesh collector) and mixed inoculum in this study showed certain members of the bacterial electrode community increased in relative abundance from the initial inoculum. For example, *Proteobacteria* species are important for electricity generation in the anode biofilms and *Firmicutes* phylum was predominant on anolyte to transfer electron. Likewise, the biofilm can act as a barrier which reduces diffusion of the anolyte towards the electrode, all the while behaving like a porous material. The anolyte and its interaction with the biofilm exert a considerable influence on diffusion processes, given that it presents the highest values for Rd. Rd increased at week 20.

## Methods

### MFC preparation

**Figure 7:**
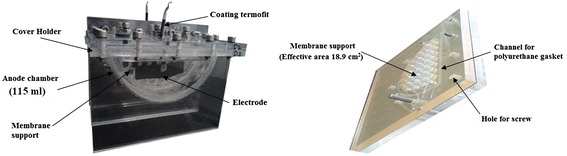
**Assembled MFC and membrane support.**

Two MFCs were constructed from acrylic. The anode and cathode chambers were semicircular in shape with a capacity of 115 ml and a volume of 115 ml (Figure 7). Nafion® 117 supported between two acrylic sheets was used as a proton exchange membrane (Figure 7). The effective area of the previously activated membrane was 18.9 cm^2^. The covers of each MFC featured ports for working, reference and counter electrodes for feeding and obtaining analysis samples, as well as for the oxygen diffuser at the cathode. The anode was carbon cloth (supplied by ElectroChem) with an area of 9 cm^2^. The cathode used 30 g granular carbon with no catalyst, using a stainless steel mesh as the current collector (mesh size 400 × 400, alloy 316). Electrode connections to the exterior were made from stainless steel mesh (mesh size 400 x 400) with nylon thread covered with Termofit® (Figure 7).

### Inoculum and carbon source

The inoculum was a mixed non-anaerobic consortium which consisted of 30 g/L deep soil, 300 g/L cattle manure, 150 g/L pig manure, 1.5 g/L sodium carbonate and 1 L water. The MFC was fed synthetic wastewater with acetate as a carbon source (6 g/L) with elements in g/L, NaHCO_3_ 1, Na_2_CO_3_ 1, KH_2_PO_4_ 0.2, NH_4_Cl 0.1 and minerals in mg L-1, ZnCl_2_ 10, CaCl_2_ 10, FeSO_4_•7H_2_O 10, CoCl_2_•6H_2_O 5, CuSO_4_•5H_2_O 5, NiCl_2_•6H_2_O 20, MnCl_2_•4H_2_O 20 [36].

### MFC configuration and operation

2 MFCs (semi-batch experiment with hydraulic retention time of 48 hours) were inoculated with 50 ml inoculum, 20 ml sodium phosphate buffer (Na_2_HPO_4_, 4.09 g/L and NaH_2_PO_4_ · H_2_O, 2.93 g/L) and 40 ml SW (Synthetic Wastewater) [37]. A control was evaluated under the same conditions.

The anode chamber was sparged with nitrogen gas to displace the oxygen present prior to closure and each time samples were taken. The pH was close to neutral. pH was adjusted with a KHCO_3_ and K_2_CO_3_ (0.2 M) buffer in accordance with Puig *et al*. [38].

In the cathode chamber a potassium phosphate buffer (50 mM) as catholyte in accordance with Zhang *et al.* [39] was used, and the pH was maintained between 4 and 5 with the use of KHCO_3_ and K_2_CO_3_ 0.2 M and bubbled with filter-sterilized air. The working temperature was 27 ± 2°C.

### Chemical analyses

The pH and temperature were measured with a Thermo Scientific Orion® multiparameter meter. COD was measured with the potassium dichromate in digestion solution technique (high range COD reagent from 0 to 15,000 ppm).

After a liquid sample was passed through a 0.22 μm pore membrane (type GV, Millipore), volatile fatty acids were analyzed using liquid chromatography. For the eluant, a mixture of an equal volume of 5 mM p-toluenesulfonic acid solution and 20 mM Bis-Tris solution containing 5 mM p-toluenesulfonic acid and 100 μM EDTA was used at 0.8 ml/min. The filtrate was acidified with concentrated HCl and short-chain alcohols [23] were analyzed using a gas chromatograph (GC - Perkin Elmer Clarus 500) with a flame ionization detector and an EC-1000 column (Altech).

Methane and hydrogen at the headspace of the anode chamber were measured using a gas chromatograph (GC - Perkin Elmer Clarus 500) equipped with a thermal conductivity detector and parallel packed columns (molecular sieve 5A) as described previously [40].

### Bacterial composition identification

The DNA extraction protocol for identifying the bacterial composition of sludge used in microbial fuel cells was used as described previously by Canto-Canché *et al*. [41]. Metagenomic DNA was sent to the Research and Testing Laboratory facility in Lubbock, Texas (USA) for pyrosequencing of the 16S rRNA gene. The Research and testing analysis pipeline performed denoising and chimera checking. Sequences of ca. 398nt were submitted for analysis. Rarefaction curves were constructed by using the tool aligner, complete linkage clustering, and rarefaction of the RDP pyrosequencing pipeline. Shannon [42] and Chao indices [43] were calculated with the complete linkage clustering data.

### EIS Experiments

Impedance measurements were taken on three different test cell configurations, as detailed in Table 2.

**Table 2:** **Different test cell configurations used for EIS studies**

**Configuration**	**Working electrode**	**Reference electrode**	**Counter electrode**	**Resistive components in the configuration**
Anode chamber	Anode	Ag/AgCl	Platinum mesh	Anode, membrane and anolyte
Biofilm (without anolyte)	Anode			Sterile buffer solution**
Anolyte (without biofilm)	Sterile Anode*			Sterile Anode*

*Carbon cloth.

**Per L of deionized water without acetate.

The EIS experiments were performed during biofilm growth at 1 and 20 weeks during closed circuit operation. Biologic potentiostat-AC frequency analyzer equipment was used for the EIS experiments, and the results were analyzed using EC-Lab® V10.23 software by χ^2^ minimization, obtaining values between 10^−2^-10^−3^. The resistance values were normalized based on the area of the electrode (9 cm^2^). The frequency of the AC signal was varied from 0.1 Hz to 1 MHz with an amplitude of 10 mV. Impedance experiments were performed under galvanostatic closed circuit conditions at 0, 50, 100, 250, and 400 mA for the immature biofilm (week 1) and at 0 and 400 mA for the developed biofilm (week 20) for comparison purposes according to the methodology of Ramasamy *et al*. [10]. To ensure steady state during galvanostatic operation, the MFC was allowed to equilibrate for 10 min between each current setting before applying the AC signal. Experiments under potentiostatic control were performed utilizing a three-electrode arrangement consisting of the working electrode, a Ag/AgCl, sat. KCl, (197 mV vs. SHE) reference electrode and a counter electrode (platinum mesh).

The equivalent circuit of the anode chamber at weeks 1 and 20 contemplates the ohmic resistance, the charge transfer process and the double layer on the electrode, as well as diffusion and transfer processes from the electrolyte towards the surface of the electrode. These were, therefore, considered resistances. The constant phase element (CPE) is attributed to the heterogeneity of either the electrode or the reactions. The controlled diffusion (W) and anomaly (M_a_) elements correspond to proton transfer processes (Rd) which, as will be explained later, are mainly attributed to the anolyte.

## Abbreviations

MFC: Microbial fuel cell
COD: Chemical oxygen demand
EIS: Electrochemical Impedance Spectroscopy
VFAs: Volatile fatty acids
SW: Synthetic wastewater
SHE: Standard hydrogen electrode
